# The Effect of Enriching Honey with Propolis on the Antioxidant Activity, Sensory Characteristics, and Quality Parameters

**DOI:** 10.3390/molecules25051176

**Published:** 2020-03-05

**Authors:** Celina Habryka, Robert Socha, Lesław Juszczak

**Affiliations:** Department of Food Analysis and Evaluation of Food Quality, Faculty of Food Technology, University of Agriculture in Krakow, Balicka 122 Str., 30-149 Krakow, Poland; celina.habryka@gmail.com (C.H.); robert.socha@urk.edu.pl (R.S.)

**Keywords:** honey, propolis, phenolic compounds, antioxidant activity, sensory characteristic

## Abstract

Bee products, including propolis, are a valuable source of biologically active substances. The most natural way to introduce propolis in the diet seems to be its addition to honey. The aim of this study was to analyze the effect of honey enrichment with propolis on the content of selected bioactive compounds, antioxidant potential, as well as sensory and qualitative characteristics of honey. On the basis of the obtained results, it was stated that the addition of propolis extract to honey contributed to a significant increase in the content of polyphenolic compounds, including flavonoids and phenolic acids, among which chrysin, pinocembrin, p-coumaric, and ferulic acid reached the highest level. The increase of antioxidant, antiradical, and reduction in activity of enriched honey was observed as a result of increasing addition of propolis extract. However, the enrichment of honey with propolis contributed to deterioration of the sensory properties. The changes in color, smell, texture, and taste were observed. The addition of propolis to honey had no significant effect on 5-hydroxymethylfurfural (HMF) and sugar content, and specific conductivity. On the other hand, a significant increase in free acidity and content of substances insoluble in water was observed. The obtained results indicate that honey supplemented with propolis extract can be an excellent source of antioxidant compounds, nevertheless, the amount of additive used is strongly dependent on changes in sensory characteristics and consumer acceptability.

## 1. Introduction

Honey and bee products are a valuable and rich source of biologically active substances [1,2,3,4]. They have been used for centuries in traditional folk medicine due to their wide spectrum of antibacterial, antiradical, antioxidant, and anticancer activity, as well as their supporting effect in prevention and treatment of many diseases [5,6,7,8]. For this reason, bee products can be attractive components of health-supporting and functional foods, due to their significant antioxidant potential [9]. Among the compounds having antioxidant properties, the most important are certain proteins and amino acids, carotenoids, phenolic compounds and flavonoids, ascorbic acid, organic acids, and Maillard reaction products [4,5,10]. The antioxidant profile of honey and other bee products and their biological activity depends on many factors, including the plant species that is the source of nectar and their varieties, as well as season of the year, climate and environmental conditions, genetic factors, and others [4,11,12].

Honey is a very valuable preventive, pro-health, and medicinal product. Honey consumption contributes to improved immunity and enriches human’s diet in many valuable nutrients and bioactive substances [13]. The bioactive substances also include polyphenols, which are organic chemical compounds naturally present in honey [5,7,8,14]. Polyphenols are important secondary metabolites of plants which are transferred to honey with nectar, pollen, or propolis. They may occur in the form of phenol glycosides, phenolic acids, free phenols, flavonoids, catechins, leucoanthocyanins, and anthocyanins [6,8]. The total phenolic content in Polish honeys ranges from 267 to 1260 mg/kg of honey [6,8,15]. The phenolic acids most commonly found in honey include: p-hydroxybenzoic, p-coumaric, cinnamic, gallic, ferulic, and caffeic acids [6,15]. They are most abundant in heather and buckwheat honeys [6,8]. The most important flavonoids found in honey are quercetin, myricetin, chrysin, apigenin, luteolin, pinocembrin, and pinobanksin [4,5,6,8,15]. Flavonoids found in nectar are transferred to honey after their proper conversion by the enzymes present in bee saliva. Some flavonoids e.g., pinocembrin, pinobaksin, and chrysin are transferred to honey with propolis in the amount dependent of its geographical origin. However, some flavonoids such as kaempferol, are known to be present in both nectar and propolis [4]. The presence of certain flavonoids in a honey is also an indicator of its geographical origin. Due to the high content of various antioxidants, honey has antioxidant properties that affect its nutritional and technological value. Antioxidants in honey are characterized by a diverse mechanism of action including reducing the negative properties of the reactive oxygen and nitrogen species, inhibiting the activity of enzymes responsible for the production of superoxide anions and chelating metal ions, as well as disrupting the radical chain reactions [16]. Honey introduced into the market must meet also the relevant quality requirements, which are governed by the Regulation of the Ministry of Agriculture and Rural Development in Poland [17], and by the relevant Directive in the European Union [18].

Of all bee products, propolis requires a special notice. It is a viscous and sticky, resin-like substance produced by bees from a mixture of insects’ secretions and resin collected from plants [10,19]. Its color can be yellow, red, green, grey-green, brown, or even black. In Poland, similarly as in other countries of Central and Eastern Europe, propolis is mainly obtained from the black poplar (*Populus nigra*) or birch (*Betula*). The origin of raw material influences not only its color, but also diverse its chemical composition [10,11,19,20,21]. In the ethanol extract of propolis, several hundred different compounds were detected and determined, with various biological activity. It included phenolic compounds, flavonoids, terpenes, lipid substances, bioelements, and minor compounds [10,20,21,22,23]. Phenolic acids and flavonoids are the main components responsible for antioxidant activity of propolis [21]. The most important phenolic acids found in this bee product are cinnamic, caffeic, and ferulic acids. The most important flavonoids are chrysin, kaempferol, apigenin, pinocembrin, and pinobanksin [10,20,24,25,26]. Besides flavonoids, the benzoic acids and numerous aromatic esters, including phenyl caffeate and benzil ferulate may also occur in this product [10,21,22]. Propolis has a strong antibacterial, anti-inflammatory, immunostimulating, chemopreventive, cytostatic, and pro-apoptotic activity [3,27,28]. Phenolic compounds found in propolis inhibit formation of amino, oxide, and peroxide radicals, as well as inhibit complexions of transition metals and free radical reactions, and also prevent lipid peroxidation [27]. Antioxidant properties of propolis are also associated with its anticancer activity, as it can inhibit proliferation of cancer cells and cause their apoptosis. Caffeic acid phenethyl ester (CAPE) is the compound of the greatest significance in cancer prevention; however, its activity depends on a synergistic effect of all the compounds present in propolis [29]. Propolis can be added to honey in the form of concentrated alcohol extract [12,30]. However, due to its intense smell and taste, the amount that can be added is rather limited. As Osés et al. [31] report, the quantity of propolis added to honey should not exceed 1% due to its adverse effect on sensory properties of honey.

Bee products are increasingly appreciated by consumers, due to their high content of bioactive compounds, among which polyphenolic compounds, being natural antioxidants, are an important group. Propolis is an abundant source of these substances. Enrichment of honey with propolis appears to be the most natural way to introduce it into a human’s diet [30,31]. However, it can be assumed that the introduction of propolis to honey will affect its sensory characteristics and parameters characterizing its commercial quality. For this reason, the aim of this work was to evaluate the influence of multifloral honey enrichment with concentrated propolis extract on a content of certain phenolic acids and flavonoids, and its antioxidant properties, sensory characteristics, and parameters determining commercial quality.

## 2. Results and Discussion

### 2.1. Influence on the Antioxidant Properties by Honey Enrichment with Propolis

Honey and propolis are the natural products having valuable nutritional, medicinal, and antioxidant properties. The antioxidant activity of these products depends on their chemical composition. Bee products contain polyphenolic compounds exhibiting a strong anti-inflammatory, biocidal, or anticancer activity, and their profile depends on the plant source [3,4,6,28]. The therapeutic effect of honey and propolis is caused mainly by their strong antiradical activity and reductive properties, contributing to deactivation of free radicals [3,4,10,32].

The total phenolic content in multifloral honeys and honeys enriched with propolis is shown in Table 1. In the analyzed honey, the content of the polyphenolic compounds was 30.75 mg GAE/100 g. This value is within a wide range of the total phenolic content in Polish multifloral honeys, as reported in the literature [8,9]. Honey enrichment with propolis in the amount from 0.1% to 1.0% raised significantly (*p* < 0.01) these compounds content from 44.46 mg GAE/100 g to 133.60 mg GAE/100 g, respectively (Table 1). Similar results were reported by Socha et al. [30], on the basis of their studies on contents of polyphenolic compounds in the commercial samples of propolis enriched honey. Also, Osés et al. [31] found that propolis addition to honey led to a significant increase in the total phenolic content, and that propolis extracts were an excellent source of various biologically active substances, including antioxidants. According to Socha et al. [20], propolis extracts were characterized by a very high content of polyphenolic compounds, within a range from 15 to 19 g/100 g.

Another important group of bioactive substances found in honey are flavonoids. They are naturally transferred by bees to honey with pollen and propolis [6]. The total content of flavonoids in multifloral honeys and in honeys enriched with propolis is shown in Table 1. The analyzed multifloral honey was characterized by the flavonoid content at a level of 2.77 mg QE/100 g. In the honeys with increasing addition of propolis, an significant (*p* < 0.01) increase in flavonoid content was noted, from 3.43 mg QE/100 g for 0.1% addition to 14.90 mg QE/100 g for 1.0% addition of propolis. Also, Osés et al. [31] determined a similar total flavonoid content in the honeys enriched with propolis. They also noted that the values obtained for enriched honeys were significantly higher than for initial honeys. A high content of flavonoids in propolis extracts [20] used to enrich samples of honey caused a significant increase in the total content of these compounds, despite the very low concentration of propolis used as an ingredient of honey.

Honey is also known as a source of phenolic acids. The honey under study, had 11.02 mg CAE/100 g of total phenolic acids. The addition of propolis, despite its low level (below 1%), significantly (*p* < 0.01) influenced the increase in the total phenolic acid content in honey. Adding 1.0% propolis, increased the honey total phenolic acids to 43.87 mg CAE/100 g (Table 1).

Anthocyanins are natural plant pigments exhibiting antioxidant activity, which are present in vacuolar fluid of flowers and fruit. In the multifloral honey, the determined anthocyanin content was in the amount of 2.01 mg/100 g (Table 1), which was within a relatively narrow range reported in the literature [33]. An addition of propolis extract resulted in a significant (*p* < 0.01) increase in the anthocyanin content from 2.73 mg/100 g for 0.1% addition of propolis to 5.25 mg/100 g at 1.0% addition of that bee product, and this indicates that propolis is also a rich source of this group of compounds.

Apart from anthocyanins, honey also contains other natural pigments, such as carotenoids, belonging to the natural antioxidants and playing an important role in a human health [12]. The total content of carotenoids in the multifloral honey was significantly lower when compared to anthocyanins, and amounted to 0.138 mg/100 g (Table 1). The addition of propolis also influenced a significant (*p* < 0.01) increase in the carotenoid content; however, it was not as high as for other bioactive substances. For a maximum level of honey enrichment of 1.0%, the carotenoid content increased to the level of 0.190 mg/100 g (Table 1).

On the basis of obtained results, correlations, well known from literature, were observed. The total phenolic content was significantly correlated with the total flavonoids (r = 0.9645), total phenolic acids (r = 0.9995), and total anthocyanins (r = 0.9817) content.

Apart from the total flavonoids content in multifloral honey and in honeys enriched with propolis, a content of certain flavonoids was also determined. Chrysin, galagin, kaempferol, and quercetin were found in all the samples, while honeys enriched with propolis also contained pinobanksin and pinocembrin (Table 2). According to the literature data, chrysin, quercetin, kaempferol, apigenin, and galangin are the most commonly flavonoids found in Polish honeys [8,10,30]. They have a strong antioxidant activity and affect color of honeys. In the studied multifloral honey, kaempferol was found to be the most abundant among all the determined flavonoids, and its content was 0.049 mg/100 g (Table 2). The second most abundant flavonoid found in the multifloral honey was quercetin, whose content was determined in the amount of 0.040 mg/100 g (Table 2). Furthermore, galagin and chrysin were also determined in the multifloral honey, while no pinobanksin and pinocembrin were found (Table 2).

In the honey enriched with increasing addition of propolis, an increase in contents of analyzed flavonoids was found (Table 2). The greatest increase in enriched honeys, exceeding 700 times relative to multifloral honey, to a level of 10.851 mg/100 g was observed for chrysin in the honey when 1.0% propolis was added. The addition of only 0.1% of propolis to honey resulted in a significant (*p* < 0.01) increase in content of this flavonoid to 1.121 mg/100 g. Socha et al. [30] determined a much wider range of chrysin content in the commercial honeys enriched with propolis. As Socha et al. [20] reported, Polish propolis was characterized by a high content of this flavonoid, within the range from 1.5 to 4.6 g/100 g. The high chrysin content in propolis was also determined by Escriche and Juan-Borras [23]. For this reason, even the small addition of propolis results in a significant increase in content of this flavonoid. Propolis addition to honey also resulted in a significant (*p* < 0.01) increase in galangin content. Its level ranged from 0.334 mg/100 g for 0.1% addition of propolis to 3.414 mg/100 g for the honey containing 1.0% of this substance (Table 2). The obtained results are within the range reported by Socha et al. [30] for the commercial samples of propolis enriched honey. According to Gargoun [29] and Osés et al. [34], galangin is one of the main flavonoids found in propolis. Also, in the case of two remaining flavonoids, i.e., kaempferol and quercetin, a significant (*p* < 0.01) increase in their content was noted with an increasing propolis concentration in honey, and this also results from a lower content of these flavonoids in honey. Both Socha et al. [20] and Escriche & Juan-Borras [23] found that quercetin levels in propolis were significantly lower when compared with other flavonoids.

Pinobanksin or pinocembrin were not found in the analyzed multifloral honey. However, addition of 0.1% propolis contributed to significant (*p* < 0.01) increase in pinobanksin content to 0.402 mg/100 g (Table 2), and 1.0% addition of propolis further raised its value to 1.867 mg/100 g. According to Mavri et al. [35], pinobanksin and pinobanksin-3-O-acetate are two of the most important flavonoids found in propolis. The pinocembrin content increased significantly (*p* < 0.01) from 0.875 mg/100 g with 0.1% addition of propolis to 8.371 mg/100 g for 1.0% propolis content in the honey. Although both discussed flavonoids are not characteristic for honeys, their presence in propolis is common [23,34,35].

**Figure 1:**
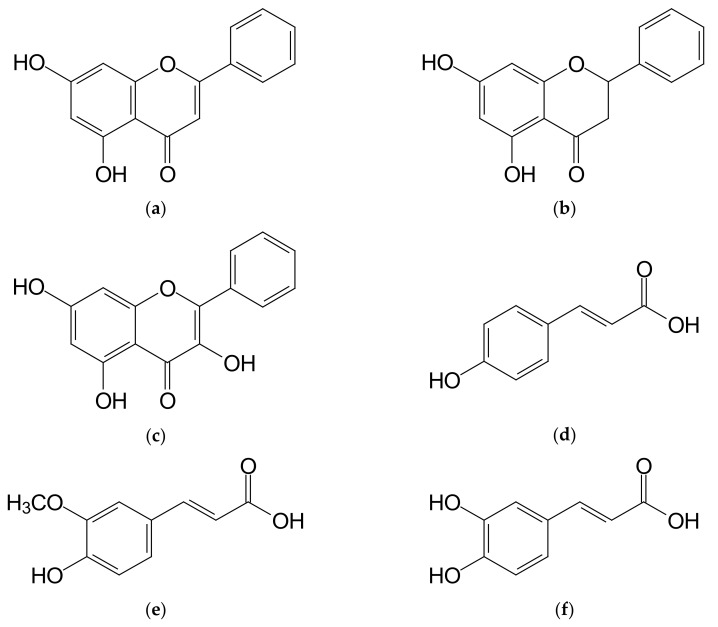
The main phenolic compounds determined in honey samples enriched with propolis. (**a**) Chrysin; (**b**) Pinocembrin; (**c**) Galangin; (**d**) p-Coumaric acid; (**e**) Ferulic acid; (**f**) Caffeic acid.

Results of determinations of individual phenolic acids contents in the multifloral honey and in honeys enriched with propolis are shown in Table 3. The analyzed multifloral honey did not contain chlorogenic acid, and this confirms the data from earlier reports for Polish honeys [9,30]. A small, even 0.1% addition of concentrated propolis extract contributed to a significant (*p* < 0.01) increase in chlorogenic acid content in the honey to a level of 0.063 mg/100 g (Table 3), and further addition of propolis resulted in an significant (*p* < 0.01) increase in chlorogenic acid content. This observation confirms data obtained by Socha et al. [30] for the commercial samples of propolis enriched honey. Aliyazıcıoglu et al. [22] found the presence of chlorogenic acid in propolis in the quantity ranging from 1.88 mg/100 g up to 160.0 mg/100 g, indicating that this bee product is a rich source of this acid. Ferulic acid content in the multifloral honey amounted to 0.095 mg/100 g (Table 3), and this confirms previous literature data [9,30]. With an increasing amount of propolis added to honey, a high, 140-fold and significant (*p* < 0.01) increase in ferulic acid content was noted for 1.0% addition of that substance. The content of this acid increased within the range from 1.438 mg/100 g (0.1%) to 13.637 mg/100 g (1.0%). According to Socha et al. [20], Polish propolis contains from 1.9 to 4.4 g/100 g of ferulic acid. Gallic acid was found to be present at the highest level among all phenolic acids analyzed in the studied multifloral honey. Its content amounted to 0.217 mg/100 g of honey (Table 3). As Socha et al. [9,30] report, gallic acid is a prevalent phenolic acid in Polish multifloral honeys. An addition of propolis extract to honey contributed to an increase in contents of this acid in a range from 1.545 to 2.494 mg/100 g (Table 3). A similar gallic acid content in the commercial samples of propolis enriched honey was previously found by Socha et al. [30]. Aliyazıcıoglu et al. [22] and Socha et al. [20] reported that propolis contained from 0.87 to 38.0 mg/100 g of gallic acid. The analyzed multifloral honey contained 0.040 mg of p-hydroxybenzoic acid per 100 g (Table 3). A variable addition of propolis extract to multifloral honey led to a significant (*p* < 0.01) increase in contents of p-hydroxybenzoic acid in the range from 0.284 to 0.576 mg/100 g for 1.0% addition. Aliyazıcıoglu et al. [22] and Socha et al. [20] determined the p-hydroxybenzoic acid content in propolis at a level up to 210 mg/100 g, indicating that propolis is a very valuable source of this compound. Caffeic acid is a phenolic acid found at the lowest level in the studied multifloral honey. Its content was determined to be at a level of 0.026 mg/100 g (Table 3), confirming the previous literature data [9,30]. The multi-fold increase in this acid content was observed when honey was enriched with an increasing addition of propolis. Already a 0.1% addition of propolis resulted in a significant (*p* < 0.01) increase in this acid content by about 16 times, to 0.418 mg/100 g, while the maximum, 1.0% addition of propolis, resulted in a significant (*p* < 0.01) increase in caffeic acid content by about 100 times, to a level of 2.670 mg/100 g. The presence of caffeic acid in propolis extracts was already noted in previous studies [20,22,23,35]. p-Coumaric acid in the multifloral honey was determined in the quantity of 0.136 mg/100 g (Table 3), and this confirms previous information [9,30]. Among determined phenolic acids found in honey enriched with propolis, p-coumaric acid had the highest share. Already, a 0.1% addition of propolis to honey contributed to nearly 30-fold significant (*p* < 0.01) increase in its content to a level of 3.716 mg/100 g, and 1% addition of propolis led to an increase in contents of this acid to a level of 24.44 mg/100 g. A high content of p-coumaric acid, within a range from 0.234 to 30.283 mg/100 g, was also determined by Socha et al. [30] for the commercial samples of honeys enriched with propolis. According to Socha et al. [20], the p-coumaric acid content in Polish propolis ranges from 38 to 117 mg/g. Lower p-coumaric acid contents in propolis were reported by Aliyazıcıoglu et al. [22] and Escriche and Juan-Borras [23], and this confirms the observation that the phenolic acids content depends on the geographical origin of propolis. However, Marvi et al. [35] observed that p-coumaric acid is the main phenolic acid present in Slovenian propolis. Protocatechuic acid content in the multifloral honey was determined at a level of 0.070 mg/100 g (Table 3). Protocatechuic acid is not a common phenolic acid found in honey. Cheung et al. [36] found its presence only in seven out of forty samples of the commercial honey. However, the latter authors reported a higher content of this compound when compared with our results. While Can et al. [37] found the presence of protocatechuic acid in the range from 0.028 to 918 mg/100 g for more than a half of the analyzed samples of propolis originating from different regions of Azerbaijan. An increasing addition of propolis extract only slightly influenced an increase in its levels in honey, which ranged from 0.094 to 0.179 mg/100 g depending on the amount of propolis. The chemical structures of predominant phenolic compounds are shown in Figure 1.

An increase in content of antioxidant compounds results in an increase in antioxidant capacities. The total antioxidant activity of multifloral honey and honeys enriched with propolis is shown in Table 4. The multifloral honey was characterized by total antioxidant activities amounting to 9.24 mM AAE/100 g. Despite the small addition of propolis used, the activity of enriched honey increased significantly (*p* < 0.01) to the level of 10.69 mM AAE/100 g already for an addition of 0.1%. The highest value of total antioxidant activity equal to 15.34 mM AAE/100 g was found of honey with 1.0% addition of propolis (Table 4). The antiradical activity of multifloral honey measured in a reaction with ABTS (2,2’-azino-bis(3-ethylbenzothiazoline-6-sulfonic acid) radical cation of multifloral honey was determined at a level of 1.78 mM TE/100 g. This activity was found to be at a level from 2.96 to 14.28 mM TE/100 g for the honey with propolis added in the range from 0.1% to 1.0% (Table 4). An addition of propolis contributes to the total content of polyphenolic compounds (Table 1), flavonoids and phenolic acids (Table 2 and Table 3), and this translates to a significant (*p* < 0.01) increase in antiradical properties. The antiradical activity of multifloral honey and honeys enriched with propolis was determined in a reaction with the DPPH (2,2-diphenyl-1-picrylhydrazyl) radical (Table 4). The antiradical activity measured using the DPPH assay for the multifloral honey was 0.26 mM TE/100 g. It was also found that this activity increased, despite a low propolis concentration added to honey. For 0.1% addition of propolis, the significant (*p* < 0.01) increase in its activity was determined at a level of 0.57 mM TE/100 g, while this activity increased significantly (*p* < 0.01) to 2.34 mM TE/100 g. Also, Socha et al. [30] and Osés et al. [31] observed a rising antiradical activity of the honey samples with propolis addition, in comparison to the honey that has not been enriched.

Reductive capacity of the multifloral honey against Fe(III) ions was determined at a level of 233.9 μM Fe(II)/100 g (Table 4), and the obtained results were within a wide range of values reported in the literature [1,38]. The propolis addition increased significantly (p < 0.01) the reductive capacity of honey within a range of 309.9 and 1038.6 μM Fe(II)/100 g (Table 4). The reductive capacity of analyzed honeys was also determined using the CUPRAC (CUPric Reducing Antioxidant Capacity) method. The reductive capacity of multifloral honey was 77.81 μM TE/100 g (Table 4). Similarly, as for the FRAP (Ferric Reducing Antioxidant Power) method, an increasing amount of propolis extract added to honey caused a significant (*p* < 0.01) increase in the reduction capacity from 181.96 to 531.54 μM TE/100 g (Table 4). Due to high quantities of polyphenolic compounds (flavonoids, and phenolic acids in particular) present in honey with propolis, despite a small addition of this substance, the reductive capacity of analyzed samples increased significantly (*p* < 0.01).

A proportional increase in the content of bioactive substances, especially flavonoids and phenolic acids, caused by an addition of propolis extract (Table 1) led to an increase in antioxidant and antiradical activities, and in a reductive capacity (Table 4). Therefore, the following correlations known from the literature were observed. The total phenolic content positively correlated with a total antioxidant activity (r = 0.9665), an antiradical capacity against ABTS^•+^ (r = 0.9879) and DPPH^•^ (r = 0.9979), and reductive capacity determined with the FRAP (r = 0.9947) and CUPRAC (r = 0.9909) methods. Similar high correlations were observed for the total flavonoid content (r = 0.9761; 0.9738; 0.9755; 0.9831; 0.9626, respectively) and the total content of phenolic acids (r = 0.9731; 0.9893; 0.9992; 09968; 0.9917, respectively), and parameters characterizing antioxidant properties. Similarly significant, although slightly lower correlations were observed for the total content of anthocyanins and carotenoids.

### 2.2. Sensory Characteristics of Honey with Propolis

**Figure 2:**
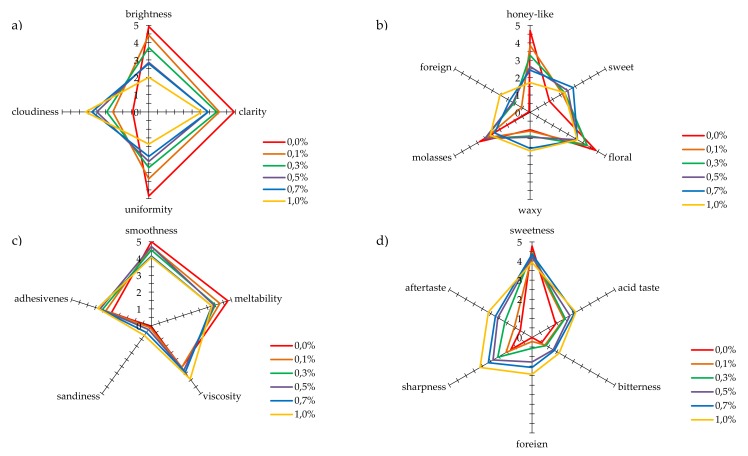
The results of sensory profiling analysis for: (**a**) color, (**b**) smell, (**c**) texture, and (**d**) taste.

As propolis, due to its specific sensory properties, also modifies properties of honey to which it is added, a detailed analysis by sensory profiling of four basic parameters: color, smell, texture, and palatability, was also performed (Figure 2). The following descriptors: brightness, uniformity, clarity, and cloudiness, were used to evaluate the color. The studied multifloral honey not enriched with propolis was assessed as very bright, highly uniform, and clear. Furthermore, the conducted analysis did not show its clouding (Figure 2a). The sensory panel indicated that a positive perception of brightness, uniformity, and clarity of honeys decreased rising addition of propolis. The perception of the cloudiness of evaluated samples went in the opposite direction, increasing as the propolis addition raised.

To evaluate the smell, the following descriptors were selected as relevant: honey-like, waxy, sweet, molasses, floral, and foreign. The sensory panel described the smell of multifloral honey that has not been enriched as strongly perceptible, floral, and honey-like. The intensity of the sweet smell impression was described as barely perceptible. The waxy and molasses smells in the studied honey were described as barely and moderately perceptible, respectively, and a foreign smell was not noticeable. The perceptibility of the foreign smell was most strongly influenced by 1.0% addition of propolis, and the sensory panel described it as hardly perceptible (Figure 2b). The increasing addition of propolis most strongly influenced the perception of intensity of honey like smell. This smell for honey without additives was evaluated as very strongly perceptible. With an increase in the propolis level in the evaluated samples, the intensity of perception of this smell decreased down to hardly perceptible. With the increase in the propolis content, the impression of molasses and wax smells was hardly perceptible for the sensory panel. The sweet smell, on the other hand, was described as moderately perceptible for a 0.7% addition of propolis.

The next characteristic of honeys with propolis added was their texture. The assessors specified the level of intensity of an impression of: smoothness, meltability, viscosity, sandiness, and adhesiveness. The texture of multifloral honey was characterized by strongly perceptible smoothness and meltability. This honey was moderately viscous and adhesive, while sandiness of this sample was described as unnoticeable (Figure 2c). The increasing addition of propolis to honey influenced the sensory evaluation of the honey texture. The perceptibility of smoothness and meltability slightly decreased with the amount of propolis added, although with the highest addition, these distinctions were assessed as strongly perceptible. In the case of the perception of viscosity and adhesiveness, the addition of propolis caused a slight increase in the intensity of feeling these characteristics. The propolis addition to honey did not significantly influence an increase in perceived sandiness. According to the sensory panel, the intensity of perceived sandiness remained at an unnoticeable level.

In an overall assessment of taste, multifloral honey was evaluated as very sweet. Acid, bitter, and sharp tastes, and remaining aftertaste were all described as barely perceptible or at a threshold level. The perceptibility of a foreign taste intensity was evaluated as unnoticeable (Figure 2d). The propolis addition to honey modified the product taste to some extent. For the 1.0% addition of propolis, the perceptibility of sweet taste went down from very strong to strong, while the perception of acid and sharp taste, and aftertaste left increased to the moderate level. Bitter and foreign tastes were barely noticeable in honeys with 0.3% addition of propolis, while the intensity of these characteristics was slightly higher for honeys with 0.5% to 1.0% addition, and the evaluators indicated that they were slightly noticeable.

**Figure 3:**
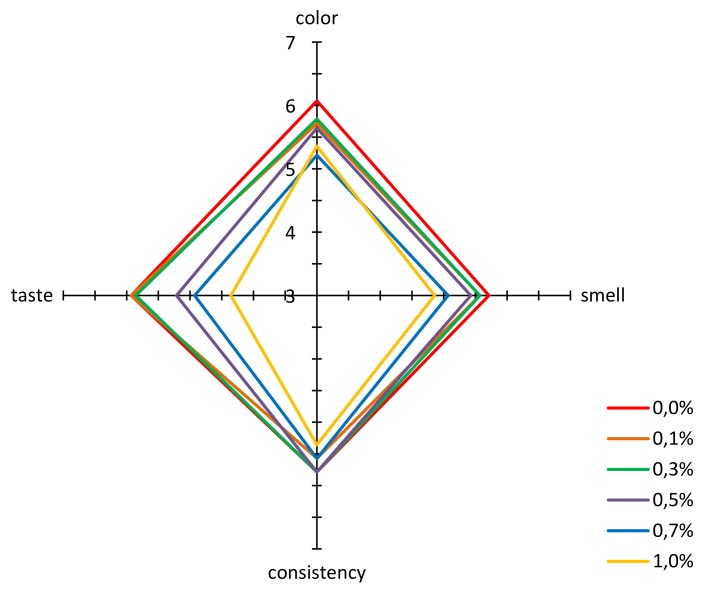
Results of the acceptability evaluation for multifloral honey and honeys enriched with propolis.

The acceptance according to the hedonic scale was also evaluated for propolis enriched honeys. For all the evaluated parameters, i.e., color, smell, texture, and palatability, the multifloral honey not enriched with propolis achieved a high acceptability at a level of 7 points, to which the wording “I like very much” corresponds (Figure 3). An addition of propolis resulted in a decrease in acceptability of honey color, resulting from an increase in cloudiness and a decrease in clarity and transparency (Figure 2a). However, the color of honey even with the highest addition of propolis was acceptable at a level above 5 points, to which the description “neither like nor dislike” corresponds. The situation was similar for texture. An increasing propolis addition to honey, especially below 0.5%, did not significantly influence the acceptability of this characteristic, and for the highest propolis addition, acceptability of the sample scored above 5 points.

An addition of propolis extract to honey at a level of up to 0.5% did not have a great influence on acceptability of smell. A significant drop in this acceptability of smell was observed for samples with a higher content of propolis, i.e., 0.7% and 1.0%. These observations confirm the results obtained with the sensory profiling method (Figure 2a), where it was found that addition of propolis clearly deteriorated the perceptibility of the honey note and increased the intensity of foreign smell.

An addition of propolis extract to honey had a varying influence on its taste. With the propolis concentration at a level of 0.1% and 0.3%, the taste acceptability was practically identical as for the honey that had not been enriched (Figure 3). Only higher concentrations of propolis caused a significant drop in acceptability, below 5 points, for concentrations of 0.7% and 1.0%. As it is shown in Figure 2d, propolis added to honey at an increasing concentration led to a significant increase in sharp, bitter and foreign taste, and presence of aftertaste.

### 2.3. Propolis Influence on Honey Quality Parameters

In Polish legislation, the specific requirements for commercial quality of honey are governed by the Regulation of the Minister of Agriculture and Rural Development [17], as amended. These regulations, consistent with Council Directive (EC) [18] specify threshold values for physical and chemical parameters crucial for the commercial quality of honey.

The water content in honey is an important parameter determining its quality. When it is too high, it can negatively affect honey stability, including its susceptibility to the fermentation processes, the activity of osmophilic yeast, and certain physical and chemical properties. The maximum acceptable water content in honey is 20%, excluding the heather honey [17]. In the studied multifloral honey, the water content was 17 g/100 g of honey, and the propolis addition did not significantly (*p* > 0.05) influence this parameter (data not presented). Another parameter characterizing the commercial quality of honey is the content of water-insoluble substances, including pollen or bee bread grains, propolis, fragments of bees and other insects, bacterial spores, algal, and fungal cells. The analyzed multifloral honey contained 0.06 g of water insoluble substances per 100 g of the product, and thus meeting the legislative requirements [17]. The propolis addition to honey significantly (*p* < 0.01) increased the content of water-insoluble substances above the acceptable threshold of 0.1 g/100 g of the product, however, the amount of propolis added did not have a significant influence here (Table 5). Juszczak et al. [39] found that the addition of bee products including pollen, bee bread, propolis, or royal jelly to honey, due to their poor solubility in water, had a significant influence on the content of water-insoluble substances. The quoted authors report that supplementation of multifloral honey with bee products may result even in a 4-fold increase in the content of insoluble substances.

Honey also contains mineral compounds that affect electrical conductivity of the honey solution. The quality requirements for nectar honeys conductivity, specify a maximum value of 0.8 mS/cm [17]. The determined electrical conductivity of the studied multifloral honey was 0.50 mS/cm. An increasing addition of propolis in honey did not result in a change in the specific conductivity value (Table 5), because the addition of this bee product was low, and this observation is also confirmed by data available in the literature [39]. An increasing addition of propolis in multifloral honey also had a small influence on an increase in acidity of samples, also due to its small addition. The free acidity value rose from 24.63 mval/kg for the 0.1% to 30.93 mval/kg for the 1% addition of propolis extract, and this does not exceed the value of 50 mval/kg specified in legislation [17] and confirms earlier reports [39].

The total fructose and glucose content in nectar honey cannot be lower than 60 g/100 g of honey, while the saccharose content should not exceed 5 g/100 g. The total content of glucose, fructose and saccharose in multifloral honey and in honeys enriched with propolis is shown in Table 5. In the studied multifloral honey, the content of glucose, fructose, and saccharose was determined in the amount of 25.53 g/100 g, 39.62 g/100 g, and 1.8 g/100 g, respectively (Table 5), and this is consistent with legal requirements [17]. According to Kędzia [10], the sugar content in propolis is negligible. This fact and a small addition of propolis lead to a lack of noticeable changes in the saccharides content with an increasing addition of propolis.

Another factor determining the quality of honey is the presence of 5-hydroxymethylfurfural (5-HMF), and its increased content indicates that honey was stored in an inappropriate conditions, especially at an increased temperature. In the studied multifloral honey, the determined HMF content was at a level of 10.76 mg/kg, and this meets legal requirements [17]. An addition of propolis did not significantly influence the HMF content, and this confirms previous results for samples of propolis enriched honey [39]. Summing up the influence of propolis addition to honey on its commercial quality, it was found that it only had a significant influence on an increase in the content of water-insoluble substances, whose values exceeded the level acceptable for honey.

## 3. Materials and Methods

### 3.1. Materials

The multifloral honey (District Beekeeping Cooperative „Pszczelarz”, Krakow, Poland) and concentrated propolis extract (Biopharmaceutical Laboratory „Arria”, Krakow, Poland) were used as the experimental materials. On the basis of the preliminary sensory assessment and literature data [31] proposed maximum amount of propolis used as an additive to honey was no higher than 1%. For this reason, the honey samples were enriched with propolis in the amount of: 0.1%, 0.3%, 0.5%, 0.7%, and 1.0% per mass of honey. The tested samples were prepared in an amount of 600 g by adding concentrated propolis extract in the appropriate amount and mixing thoroughly. The samples prepared in this way were stored at room temperature in glass containers until analysis.

### 3.2. Determination of Phenolic Profile and Antioxidant Properties

Evaluation of the antioxidant properties of multifloral honey and honey samples enriched with propolis was made using the ethanolic-water solutions obtained from these products with initial concentration equal to 0.2 g/mL by dissolving the appropriate amount of sample in an ethanol-water mixture (50:50 *v/v*). Spectrophotometric analysis was carried out on an UV/Vis V-630 spectrophotometer (Jasco, Japan).

The total phenolic content was determined in the reaction with Folin-Ciocalteu reagent (F-C) following the procedure reported by Singelton and Rossi [40]. The calibration curve was plotted for standard solution of gallic acid (Sigma-Aldrich, Germany) in concentration range of 0–100 µg/mL. The absorbance measurements were conducted at λ = 760 nm. The total phenolic content was expressed as gallic acid equivalents (GAE) in mg GAE/100 g of sample.

The total flavonoid content was established in the reaction with aluminum chloride using the method described by Ardestani and Yazdanparast [41]. The calibration curve was plotted for standard solution of quercetin (Sigma-Aldrich, Germany) in concentration range of 100–500 µg/mL. The absorbance measurements were conducted at λ = 510 nm. The total flavonoid content was expressed as quercetin equivalents (QE) in mg QE/100 g of sample.

The total phenolic acids content was established in the reaction with Arnov’s reagent using the procedure described by Nalewajko-Sieliwoniuk et al. [42]. The absorbance measurements were conducted at λ = 490 nm. The total phenolic acids content was expressed as caffeic acid equivalents (CAE) in mg CAE/100 g of sample.

The total anthocyanins content was determined using the method described by Rababah et al. [33]. The absorbance measurements were conducted at λ = 675 and λ = 530 nm. The total anthocyanins content was expressed as cyanidin-3-glucoside equivalents (CGE) in mg CGE/100 g of sample.

The total carotenoids content was established using the spectrophotometric method described by Boussaid et al. [14]. The calibration curve was plotted for standard solution of β-carotene (Sigma-Aldrich, Germany) plotted in concentration range of 0.2–2.0 µg/mL. The absorbance measurements were conducted at λ = 450 nm. The total carotenoids content was expressed as β-carotene equivalents (β-CE) in mg of β-carotene/100 g of sample.

The polyphenolic profile of samples under study was conducted using a high-performance liquid chromatography (HPLC). Phenolic compounds were extracted from the studied samples using ethyl acetate as an extrahent following the procedure described by Socha et al. [15]. The qualitative and quantitative analysis of flavonoids and phenolic acids present in investigated samples was conducted with the use of a high-performance liquid chromatography (HPLC LC-Net II/ADC, Jasco, Japan) equipped with a DAD detector (MD—2018 plus, Jasco, Japan). The analysis was carried out on a Purospher RP-18 column (250 mm × 4 mm, 5 µm, Merck, Germany) at a temperature of 30 °C and at a flow rate of 1 mL/min. The solvent system was a linear gradient with two phases: phase A: aqueous solution of acetic acid (0.4 M) and phase B: acetonitrile (100%). The chromatographic analysis was conducted as follows: for the first 10 min—linear gradient with phase B contribution increasing from 3% to 8%, followed by an increase in phase B contribution to 15%, 20%, 30% and 40% at 20, 30, 40 and 50 min. The qualitative analysis of phenolic compounds was made by comparison of the UV spectra obtained for the analyzed compounds with those for the phenolic standards using the DAD detector. The quantitative analysis of analyzed phenolics was made on a base of calibration curves which were plotted separately for each standard (flavonoids and phenolic acids Sigma-Aldrich, Germany) in concentration range of 0.02–0.2 mg/mL.

Determination of total antioxidant capacity was made according to the method described by Prieto et al. [43]. The calibration curve was made in concentration range of 0.5–0.3 µM using ascorbic acid (Avantor-POCh, Poland) as a standard. The absorbance measurements were conducted at λ = 695 nm. The results were expressed as ascorbic acid equivalents (µM AAE/100 g).

Determination of antioxidant activity in the reaction with DPPH radical (Sigma-Aldrich, Germany) was made according to the method described by Blois et al. [44]. The calibration curve was made in concentration range of 0–0.09 µM using trolox (Sigma-Aldrich, Germany) as a standard. The absorbance measurements were conducted at λ = 515 nm. The results were expressed as µg of trolox per 100 g of sample (µM TE/100 g).

Determination of antioxidant activity in the reaction with ABTS cation radical (Sigma-Aldrich, Germany) was made according to the method described by Baltrušaityte et al. [45]. The calibration curve was made in concentration range of 0–0.09 µM using trolox (Sigma-Aldrich, Germany) as a standard. The absorbance measurements were conducted at λ = 734 nm. The results were expressed as µg of trolox per 100 g of sample (µM TE/100 g).

The ferric reducing ability was determined using FRAP method according to the method described by Benzie et al. [46]. The calibration curve was made in concentration range of 200–1200 µM Fe(II) using ferrous sulphate solution as a standard (Avantor-POCh, Poland). The absorbance measurements were conducted at λ = 593 nm. The results were expressed as µM of Fe(II) ions per 100 g of sample (µM Fe(II)/100 g).

The cupric reducing ability was determined using CUPRAC method in the reaction with neocuproine (Sigma-Aldrich, Germany) according to the method described by Apak et al. [47]. The calibration curve was made in concentration range of 0–0.09 µM using trolox as a standard (Avantor- POCh, Poland). The absorbance measurements were conducted at λ = 450 nm. The results were expressed as µM of trolox per 100 g of sample (µM TE/100 g).

### 3.3. Evaluation of Sensory Characteristics

The sensory analysis of honey supplemented with propolis was conducted on the basis of assessments carried out by a 14-person sensory panel with proven sensory sensitivity and trained [48] to make assessments in a sensory laboratory complying with Polish Standard [49]. The sensory analysis included the quantitative description method and assessment of acceptance using a hedonic scale [50,51]. The research methodology included determination of color, smell, texture, and taste of the multifloral honey and honey samples enriched with propolis. Intensity of perception was rated on a scale: 0 (imperceptible); 1 (barely perceptible); 2 (hardly perceptible); 3 (moderately perceptible); 4 (strongly perceptible); 5 (very strongly perceptible). In regard to color determination, the following descriptors were included: brightness, clarity, uniformity, and cloudiness. Among the descriptors used for smell assessment, the following were evaluated: honey-like, sweet, floral, waxy, molasses, and foreign smell. Texture was determined taking into account: smoothness, viscosity, adhesiveness, meltability, and sandiness. The descriptors specifying tastiness included: sweetness, bitterness, sharpness, acid taste, foreign taste, and aftertaste—persistence.

In order to analyze acceptance of the honey samples enriched with propolis, the seven-point hedonic scale was used. Color, smell, consistency, and taste were evaluated during sensory analysis. The scores were rated on a scale from 0 (I dislike very much) to 7 (I like very much).

#### 3.3.1. Physicochemical characteristics

The qualitative parameters of multifloral honey and honeys enriched with propolis were determined in agreement with the requirements of the Polish Ministry of Agriculture and Rural Affairs of 14 January 2009 [52].

Determination of the sugar (glucose, fructose, and saccharose) content was made by a high-performance liquid chromatography (HPLC) and a refractometric detection (HPLC-RI). Chromatographic analyses were made using a liquid chromatography (HPLC-LaChrom D-7000, Merck-Hitachi, Japan) with a Purospher NH2 column (250 mm × 4 mm, 5 µm). As a mobile phase, a mixture of acetonitrile with water (80:20, *v/v*) was used with a flow rate of 1ml/min. The column was thermostated at 30 °C, and the volume injected was equal to 20 µL.

Determination of 5-hydroxymethylfurfural (HMF) was made by an reversed phase high-performance liquid chromatography and UV detection (RP-HPLC-UV). Chromatographic analyses were made using a liquid chromatography (HPLC-LaChrom D-7000, Merck-Hitachi, Japan) with a Perkin Elmer Brownlee Analytical C18 column (150 mm × 4.6 mm, 5 µm). As a mobile phase, a mixture of methanol with water (10:90, v/v) with a flow rate of 1 mL/min. The column was thermostated at 30 °C, and the volume injected was equal to 20 µL. HMF was detected at λ = 285 nm.

Determination of a water insoluble matter content was made by a gravimetric method. Determination of a specific conductivity was made using a conductometer CPC 501 (Elmetron, Poland). Free acidity was determined by a titration method using TitroLine easy titrator (Schott, Germany).

#### 3.3.2. Statistical analyses

Data are expressed as mean ± SD and each value is representative of at least three repetitions. In order to establish the statistical differences between means, the dates were treated by one-factor analysis of variance, and the Least Significant Difference (LSD) values were calculated using Fisher LSD test at significance level 0.05. In addition, linear correlation coefficients were calculated between the selected variables, and their significance was verified at 0.05. Calculations were performed with statistical software package Statistica 11.0 (StatSoft Inc., Tulsa, OK, USA).

## 4. Conclusions

The study analyzed the influence of honey enrichment with ethanol extract of propolis on contents of certain bioactive compounds, antioxidant potential, and sensory and qualitative characteristics. The results obtained in the study imply that an addition of propolis extract to honey contributed to a significant increase in the content of polyphenolic compounds, including flavonoids and phenolic acids, of which chrysin, pinocembrin, p-coumaric acid, and ferulic acid were present in the highest amounts. The addition of propolis extract and an increase in contents of polyphenolic compounds resulted in an increase in antioxidant, antiradical, and reductive activities. However, honey enrichment with propolis extract resulted in its deteriorated sensory properties. For color, a drop in brightness, clarity, and uniformity were noted, accompanied by an increase in cloudiness. In taste characteristics, an increased perception of waxy, molasses, and foreign tastes was found. For the texture evaluation, a decrease in smoothness and meltability was observed, accompanied by a slight increase in viscosity and adhesion. The evaluation of taste showed a clear increase in sharp acid and bitter taste with a persistent aftertaste. The propolis addition to honey influenced an increase in free acidity and content of substances insoluble in water. The obtained results show that honey enriched with propolis extract can be an excellent source of antioxidants; however, the use of this additive strongly depends on changes in sensory characteristics and consumer acceptance.

## Figures and Tables

**Table 1:** The total phenolic, flavonoid, phenolic acids, anthocyanins, and carotenoids content in multifloral honey and honeys enriched with propolis.

Addition of Propolis (%)	Total Phenolic Content (mg GAE/100 g)	Total Flavonoid Content (mg QE/100 g)	Total Phenolic Acids Content (mg CAE/100 g)	Total Anthocyanins Content (mg/100 g)	Total Carotenoids Content (mg/100 g)
0	30.75 ± 0.25	2.77 ± 0.29	11.02 ± 0.68	2.01 ± 0.05	0.138 ± 0.001
0.1	44.46 ± 0.24	3.43 ± 0.13	15.30 ± 0.49	2.73 ± 0.06	0.164 ± 0.002
0.3	70.29 ± 0.43	8.56 ± 0.37	23.74 ± 0.70	2.88 ± 0.10	0.176 ± 0.001
0.6	88.25 ± 0.20	10.85 ± 0.42	30.12 ± 0.52	3.97 ± 0.19	0.181 ± 0.002
0.8	98.30 ± 1.41	14.02 ± 0.07	33.37 ± 0.84	4.07 ± 0.22	0.186 ± 0.001
1	133.60 ± 0.55	14.90 ± 0.38	43.87 ± 0.46	5.25 ± 0.29	0.190 ± 0.001
LSD_0.05_	0.83	0.38	0.78	0.22	0.002

**Table 2:** The content of flavonoids in multifloral honey and honeys enriched with propolis.

Addition of Proplis (%)	Chrysin (mg/100 g)	Galangin (mg/100 g)	Kaempferol (mg/100 g)	Quercetin (mg/100 g)	Pinobanksin (mg/100 g)	Pinocembrin (mg/100 g)
0	0.014 ± 0.001	0.023 ± 0.001	0.049 ± 0.004	0.040 ± 0.001	-	-
0.1	1.121 ± 0.022	0.334 ± 0.023	0.111 ± 0.007	0.038 ± 0.002	0.402 ± 0.002	0.875 ± 0.025
0.3	3.394 ± 0.096	1.078 ± 0.017	0.123 ± 0.004	0.067 ± 0.002	0.788 ± 0.002	2.609 ± 0.126
0.6	5.724 ± 0.184	1.503 ± 0.068	0.172 ± 0.002	0.116 ± 0.012	1.007 ± 0.012	3.538 ± 0.158
0.8	8.195 ± 0.266	2.841 ± 0.152	0.270 ± 0.010	0.182 ± 0.006	1.394 ± 0.071	6.633 ± 0.156
1	10.851 ± 0.154	3.414 ± 0.204	0.336 ± 0.004	0.217 ± 0.016	1.867 ± 0.059	8.371 ± 0.344
LSD_0.05_	0.189	0.135	0.007	0.011	0.053	0.239

- not detected.

**Table 3:** The content of phenolic acids in multifloral honey and honey enriched with propolis.

Addition of Propolis (%)	Chlorogenic Acid (mg/100 g)	Ferulic Acid (mg/100 g)	Gallic Acid (mg/100 g)	Hydroxybenzoic Acid (mg/100 g)	Caffeic Acid (mg/100 g)	p-Coumaric Acid (mg/100 g)	Protocatechuic Acid (mg/100 g)
0	-	0.095 ± 0.005	0.217 ± 0.004	0.040 ± 0.003	0.026 ± 0.000	0.136 ± 0.006	0.070 ± 0.003
0.1	0.063 ± 0.001	1.438 ± 0.025	1.545 ± 0.058	0.284 ± 0.015	0.418 ± 0.010	3.716 ± 0.063	0.094 ± 0.003
0.3	0.069 ± 0.000	3.842 ± 0.095	1.850 ± 0.091	0.402 ± 0.008	0.993 ± 0.033	8.189 ± 0.333	0.106 ± 0.001
0.5	0.080 ± 0.002	6.089 ± 0.135	1.938 ± 0.084	0.459 ± 0.008	1.292 ± 0.047	10.976 ± 0.600	0.121 ± 0.006
0.7	0.083 ± 0.001	9.689 ± 0.296	2.293 ± 0.083	0.526 ± 0.012	1.928 ± 0.035	16.494 ± 0.444	0.148 ± 0.002
1	0.086 ± 0.001	13.637 ± 0.660	2.494 ± 0.094	0.576 ± 0.008	2.670 ± 0.051	24.440 ± 0.402	0.179 ± 0.003
LSD_0.05_	0.001	0.378	0.95	0.012	0.043	0.465	0.004

- not detected.

**Table 4:** The antioxidant, antiradical activity and reductive activity of multifloral honey and honeys enriched with propolis.

Addition of Propolis (%)	Antioxidant Activity (mM AAE/100 g)	ABTS^+^ (mM TE/100 g)	DPPH^•^ (mM TE/100 g)	FRAP (µM Fe(II)/100 g)	CUPRAC (µM TE/100 g)
0	9.24 ± 0.12	1.78 ± 0.02	0.26 ± 0.00	233.9 ± 0.7	77.81 ± 1.44
0.1	10.69 ± 0.46	2.96 ± 0.03	0.57 ± 0.01	309.9 ± 0.7	181.96 ± 1.48
0.3	11.81 ± 0.26	5.47 ± 0.05	1.06 ± 0.01	526.7 ± 0.5	301.42 ± 0.73
0.6	13.98 ± 0.19	8.28 ± 0.03	1.49 ± 0.02	701.9 ± 0.6	373.63 ± 0.61
0.8	14.61 ± 0.23	11.23 ± 0.28	1.75 ± 0.01	831.9 ± 0.6	397.28 ± 4.00
1	15.34 ± 0.40	14.28 ± 0.05	2.34 ± 0.01	1038.6 ± 0.8	531.54 ± 2.86
LSD_0.05_	0.37	0.15	0.01	0.81	2.76

**Table 5:** The qualitative parameters of multifloral honey and the honey enriched with propolis.

Propolis Addition (%)	Insoluble Matter (g/100 g)	Free Acidity (mval/kg)	Specific Conductivity (mS/cm^3^)	Glucose Content (g/100 g)	Fructose Content (g/100 g)	Saccharose Content (g/100 g)	5-HMF^1^ Content (mg/kg)
0	0.06 ± 0.01	22.87 ± 0.15	0.500 ± 0.001	25.53 ± 0.51	39.62 ± 0.91	1.83 ± 0.06	10.76 ± 0.02
0.1	0.25 ± 0.01	24.63 ± 0.25	0.495 ± 0.001	25.32 ± 0.21	37.63 ± 0.18	2.21 ± 0.12	10.68 ± 0.03
0.3	0.25 ± 0.00	26.30 ± 0.52	0.495 ± 0.002	25.12 ± 0.05	37.63 ± 0.37	2.14 ± 0.04	9.84 ± 0.02
0.6	0.25 ± 0.01	27.63 ± 0.55	0.495 ± 0.002	24.77 ± 0.05	40.27 ± 0.28	1.74 ± 0.01	10.08 ± 0.15
0.8	0.26 ± 0.00	29.07 ± 0.21	0.497 ± 0.000	25.82 ± 0.87	40.31 ± 0.33	1.58 ± 0.03	9.84 ± 0.02
1	0.26 ± 0.01	30.93 ± 0.46	0.493 ± 0.000	24.71 ± 0.07	38.38 ± 0.27	1.61 ± 0.07	9.84 ± 0.02
LSD_0.05_	0.01	0.49	0.002	0.53	1.07	0.08	0.08

1HMF – Hydroxymethylfurfural.
